# Metabolic Imaging of Advanced Basal Cell Carcinoma Treated with Sonidegib: A Retrospective Case Series Study

**DOI:** 10.3390/jcm13175087

**Published:** 2024-08-27

**Authors:** Ilaria Proietti, Luca Filippi, Oreste Bagni, Concetta Potenza

**Affiliations:** 1 Dermatology Unit “Daniele Innocenzi”, “A. Fiorini” Hospital, Via Firenze, 1, 04019 Terracina, Italy; ilaria.proietti@uniroma1.it (I.P.); concetta.potenza@uniroma1.it (C.P.); 2 Nuclear Medicine Unit, Department of Oncohaematology, Policlinico Tor Vergata University Hospital, Viale Oxford 81, 00133 Rome, Italy; 3 Nuclear Medicine Unit, “Santa Maria Goretti” Hospital, Via Antonio Canova, 04100 Latina, Italy; o.bagni@ausl.latina.it

**Keywords:** advanced basal cell carcinoma, PET/CT, metabolic imaging, quantitative parameters, oncology, hedgehog pathway inhibitors

## Abstract

**Background**: Positron emission tomography/computed tomography (PET/CT) with ^18^F-fluorodeoxyglucose (^18^F-FDG) is a firmly established tool in oncology and is gaining importance in dermato-oncology. However, its use in advanced basal cell carcinoma (BCC) is limited, with only a few case reports and a single study focused on vismodegib. This study evaluates the role of ^18^F-FDG PET/CT in advanced BCC treated with sonidegib. **Methods**: We retrospectively assessed the clinical data of patients with advanced BCC who underwent ^18^F-FDG PET/CT between January 2022 and January 2024. Inclusion criteria included histologically confirmed BCC, FDG-avid lesions on baseline PET/CT, and a minimum follow-up of 6 months. Metabolic response was assessed using the PET Response Criteria in Solid Tumors (PERCIST). **Results**: Four patients with advanced BCC treated with sonidegib were included, presenting with a total of 10 hypermetabolic lesions at baseline PET/CT. The mean interval between baseline and follow-up scans was 8.7 ± 1.6 months. According to PERCIST, two patients achieved a complete metabolic response (CMR), while the other two had stable metabolic disease (SMD). Low baseline-standardized uptake values (i.e., SUVmax, SUVmean) and reduced total lesion glycolysis (TLG) were associated with CMR. No relapses were observed during follow-up. **Conclusions**: This study suggests that ^18^F-FDG PET/CT may help identify advanced BCC patients who are likely to benefit from sonidegib treatment. Further research is needed to fully explore the potential of PET/CT in this specific clinical context.

## 1. Introduction

Basal cell carcinoma (BCC) is the most common type of skin cancer worldwide, accounting for approximately 80% of all non-melanoma skin cancers. Originating from the basal cells in the lower part of the epidermis, BCC is typically characterized by slow growth and a low propensity for metastasis [1]. However, BCC can become advanced when it invades deeper tissues or becomes unresectable due to size, location, or patient comorbidities. Advanced BCC includes either metastatic BCC (mBCC) or locally advanced BCC (laBCC). Although precise estimates of the incidence of advanced BCC are difficult to obtain, it has been reported to constitute approximately 1–10% of all BCCs, with the occurrence of mBCC being even rarer, amounting to only 0.0028–0.5% [2]. Both laBCC and mBCC present significant clinical challenges [2]. Notably, laBCC is defined by its inability to be surgically excised with clear margins without substantial morbidity or by recurrent disease after multiple surgical attempts. Conversely, mBCC involves the spread of cancer cells to distant organs, including lymph nodes, lungs, and bones [3].

Managing advanced BCC requires a multidisciplinary approach, often involving dermatologists, oncologists, radiologists, and surgeons [4]. For advanced BCC, where surgery is not feasible, alternative treatment modalities are considered. Radiotherapy is a valuable option for patients who are not candidates for surgery due to medical comorbidities or when the BCC is located in a surgically challenging area. It is particularly effective in treating primary BCC and as adjuvant therapy post-surgery for residual disease. In recent years, significant advancements have been made in the pharmacological management of advanced BCC. Hedgehog pathway inhibitors (HPIs) have revolutionized the treatment landscape [5,6]. These drugs target the aberrant Hedgehog-signaling pathway, which plays a crucial role in BCC pathogenesis. Vismodegib, the first HPI implemented for managing advanced BCC, has demonstrated efficacy in shrinking tumors and, in some cases, achieving complete remission. However, the use of HPIs is often limited by adverse effects, including muscle spasms, dysgeusia, and alopecia, which can impact patients’ quality of life [7]. In this context, identifying patients who are likely to benefit from molecularly targeted therapies is crucial for promptly switching non-responders to more effective alternative treatments. There is an unmet need for biological and imaging biomarkers suitable for prognostic stratification and response monitoring in patients with advanced BCC undergoing various therapeutic regimens.

Positron emission tomography/computed tomography (PET/CT) with ^18^F-fluorodeoxyglucose (^18^F-FDG) has a well-established role in oncology and is gaining increasing importance in dermato-oncology [8,9]. However, its application in patients with advanced BCC remains anecdotal, limited to some published case reports, with the exception of a single study conducted on patients with advanced BCC treated with vismodegib [10]. The authors found that BCC lesions exhibited increased ^18^F-FDG uptake. Additionally, a reduction in tracer uptake on follow-up PET/CT scans was associated with a more favorable outcome. Despite these initial encouraging data, the potential of metabolic imaging in the field of BCC has not been further explored.

The aim of this case series is to investigate the role of ^18^F-FDG PET/CT in patients with advanced BCC treated with sonidegib, a HPI recently introduced into clinical practice. This study particularly focuses on patient prognostic stratification and response monitoring.

## 2. Materials and Methods

### 2.1. Study Design

We carried out a retrospective analysis of clinical data from a cohort of patients with advanced BCC. This study included all patients who underwent an ^18^F-FDG PET/CT scan between January 2022 and January 2024 at our Nuclear Medicine Unit, as part of their diagnostic process. Participants were included based on the following criteria: (1) age over 18 years; (2) a histological confirmation of BCC; (3) a PET/CT scan performed before initiating the treatment regimen (PET-1) and subsequent follow-up to evaluate therapeutic response; (4) at least one FDG-positive lesion identified in the baseline PET/CT scan; (5) a minimum follow-up period of 6 months post-PET/CT, with complete and accessible medical records. The following criteria led to exclusion: (1) absence of FDG-positive lesions in the baseline PET scan; (2) lack of complete medical history or follow-up data.

This study retrospectively reviewed data collected during routine clinical practice, where the documentation for all patients under follow-up for BCC were examined. The data were anonymized and aggregated into an electronic database for further analysis. As the study utilized anonymized data obtained after patients had consented to follow-up and data collection, there was no requirement for individual informed consent. No experimental procedures, devices, or drugs were involved, and no funding was provided for this study. This study was conducted in accordance with the ethical principles outlined in the 1975 Declaration of Helsinki.

### 2.2. PET/CT Imaging Procedure

All patients underwent PET/CT scans with ^18^F-FDG in line with current imaging protocols [11]. The whole-body PET/CT was conducted from the vertex of cranium to the upper thighs, 60 min following an intravenous (i.v.) injection of 3.7 MBq/kg of ^18^F-FDG. The PET/CT examination utilized a digital Biograph Vision PET/CT system (Siemens Healthcare, Erlangen, Germany). The PET/CT scan acquisition protocol as well as the image reconstruction process have been thoroughly described in the Supplementary Materials.

### 2.3. Image Analysis

Two board-certified nuclear medicine physicians reviewed the images using Advantage 4.7 software (GE). Any area showing a tracer uptake higher than the background and not categorized as physiological was deemed potentially pathological. For each patient, sites and the number of pathological uptakes were annotated. Baseline PET/CT parameters included the number of FDG-avid lesions, mean and maximum standardized uptake value (SUVmean and SUVmax), total metabolic tumor volume (MTV), and total lesion glycolysis (TLG). Lesions were segmented using a 42% SUVmax threshold, and TLG was calculated as MTV × SUVmean. The heterogeneity index (HI) was determined by dividing SUVmax by SUVmean [12].

### 2.4. Response Assessment and Follow-Up

PET/CT scans performed after therapy (PET-2) were compared to baseline scans (PET-1) and assessed using PET Response Criteria in Solid Tumors (PERCIST) [13]. The lesion exhibiting the highest FDG uptake on both PET-1 and PET-2 was identified as the primary target. A complete metabolic response (CMR) was defined by the complete absence of all FDG-positive lesions compared to the baseline scan. For a partial metabolic response (PMR), there needed to be at least a 30% decrease in the peak standardized uptake value (SUV) adjusted for lean body mass (SULpeak) of the primary target lesion, while non-target lesions should not have shown any signs of progression. Stable metabolic disease (SMD) was defined as no change or progression, while progressive metabolic disease (PMD) was indicated by a 30% or more increase in the SULpeak of the target lesion or clear progression in non-target lesions.

### 2.5. Statistics

Continuous variables are reported as median, mean, and standard deviation (SD), whereas categorical data are presented as counts and percentages.

## 3. Results

From an initial search in our database, eight patients with BCC who underwent PET/CT were identified. However, four subjects were excluded because they had non-invasive forms and were treated with different therapies from HPI (radiotherapy, local therapy, surgery). The remaining four patients, with locally advanced BCC, treated with sonidegib (200 mg daily), were selected. Among the enrolled patients, three were male, and one was female. Of note, three out of four cases (75%) presented aggressive histotypes. The mean age was 81.2 ± 6.0.

The clinical and demographic characteristics of the selected patients are summarized in Table 1. Of note, two patients had not received BCC-specific therapy before sonidegib, while the remaining two had previously undergone loco-regional treatments.

### 3.1. Metabolic Characteristics of Lesions at Baseline

PET-1 revealed FDG-avid skin lesions in all patients. Overall, ten hypermetabolic lesions were detected; their metabolic quantitative features are reported in Table 2. The metabolic parameters resulted in (mean ± standard deviation, median): SUVmax: 9.4 ± 4.5, 7.8; SUVmean: 5.2 ± 3.2, 3.6; MTV (cc): 1.8 ± 1.7, 1.1; TLG (g/mL): 8.7 ± 6.5, 7.7.

The HI resulted in (mean ± standard deviation, median): 1.8 ± 0.3, 1.9. In the study population, no significant inhomogeneity in the intralesional distribution of the radiopharmaceutical was found, suggesting a relative metabolic homogeneity of the BCC lesion.

### 3.2. Metabolic Response and Follow-Up

**Figure 1:**
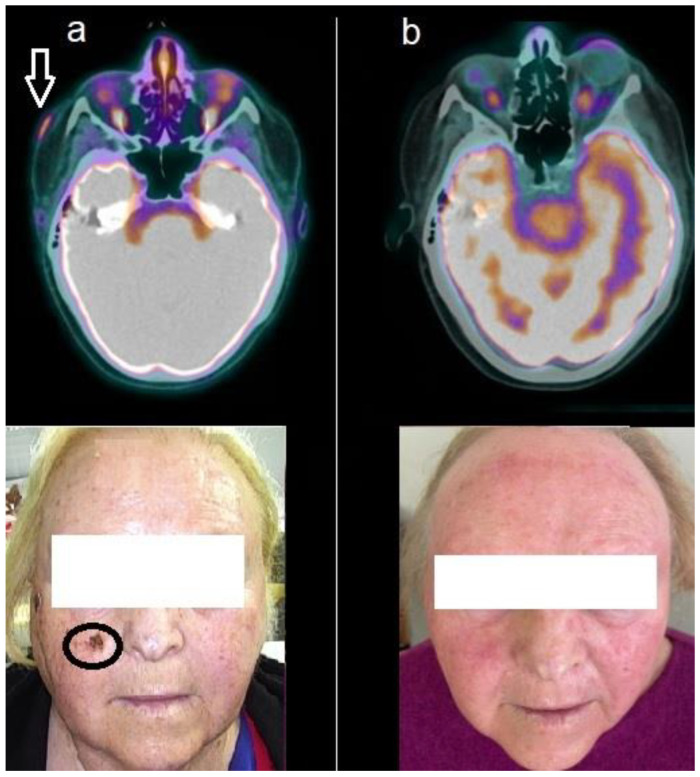
Patient n. 1, a 74-year-old woman, affected by advanced BCC. (**a**) Baseline ^18^F-FDG PET/CT showed increased tracer incorporation into the lesion of the right zygomatic region, as well evident in the fused axial (upper row, arrow) and at clinical examination (lower row, circle); (**b**) Follow-up PET/CT acquired after 8 months of HPI therapy, depicted complete metabolic response as evident in the axial images (upper row) and at clinical examination (lower row).

**Figure 2:**
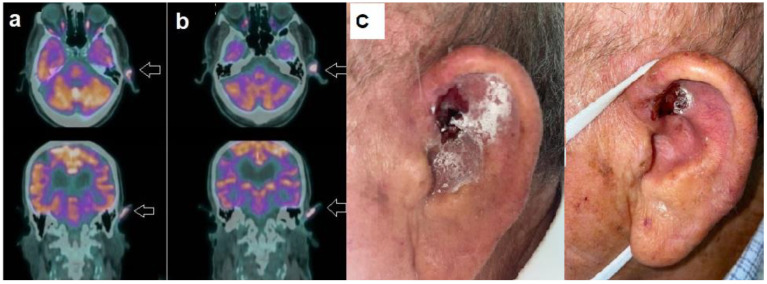
Patient n. 3, an 89-year-old-male, affected by advanced BCC. (**a**) Baseline PET/CT showed increased tracer uptake in the left ear (arrow), as well evident in the axial (upper row) and coronal (lower row) image. (**b**) Follow-up PET/CT after 8 months of HPI therapy, demonstrating stable metabolic disease according to PERCIST; (**c**) Clinical examination at the time of PET-1 (left side) and of PET-2 (right side), consistent with substantially stable disease.

The mean interval between baseline (PET-1) and follow-up scan (PET-2) was 8.7 ± 1.6 months. According to PERCIST, two patients (pt. 1, pt. 2) achieved complete metabolic response, as shown in Figure 1. Conversely, the remaining two patients showed stable metabolic response (Figure 2).

In all cases, PET-based response was in agreement with clinical and dermatoscopic evaluation. After PET-2, all patients underwent regular follow-up through laboratory tests and clinical examination for the following 6 months. No patient relapsed or progressed during follow-up. All patients are still under HPI therapy.

### 3.3. Differences in Metabolic Parameters between CMR and SMD Patients

The analysis of metabolic parameters revealed that both patients who achieved a complete response after treatment with HPI (pt. 1, pt. 2) had lower SUVmax, SUVmean, and TLG values compared to the corresponding values in patients who exhibited stable disease (pt. 3, pt. 4). In contrast, the MTV and HI parameters were not sufficiently discriminative between patients with CMR and those with SMD after HPI. Specifically, patient n. 4 had a reduced disease volume (MTV: 1 cc) and a high uptake level (SUVmax: 16.9), achieving SMD after HPI.

## 4. Discussion

Our study is the first to specifically investigate the role of ^18^F-FDG PET/CT in evaluating the therapeutic response of patients with advanced BCC treated with sonidegib. Our findings suggest that ^18^F-FDG PET/CT might help identify BCC patients likely to benefit from sonidegib treatment.

Nuclear medicine relies on the use of radiopharmaceuticals that target specific metabolic pathways or molecular markers. It is widely used to explore pathophysiological processes at both cellular and molecular levels [14,15,16]. Specifically, employing ^18^F-FDG PET/CT in patients with advanced BCC may offer significant advancements in assessing the disease’s metabolic burden and treatment response. This molecular imaging technique provides a comprehensive view of tumor physiology, which is crucial for optimizing therapeutic strategies, especially in personalized and targeted treatment contexts. In our cohort, four patients with locally advanced BCC were analyzed using various quantitative parameters. It is worth noting that both SUVmax and SUVmean, as well as TLG, have shown potential in identifying BCC patients who are more likely to benefit from sonidegib. In this context, SUV plays a well-established role in oncology for characterizing tumor biological behavior, as higher SUV levels have been correlated with more aggressive lesions [17,18]. However, SUV has several limitations, as it only provides information on the most active portion of the tumor and does not account for the overall size of the lesion. Recently, PET-derived volumetric parameters have gained increasing importance as prognostic indicators. In particular, TLG, which combines SUVmean and MTV, as well as its changes after treatment, has been found to be particularly useful in this regard [19,20]. Our findings suggest that TLG might also have potential in the field of advanced BCC undergoing molecularly targeted therapies.

We particularly focused on the HI, which measures metabolic heterogeneity within the lesion [21]. This index helps assess tumor heterogeneity, a crucial factor in understanding tumor aggressiveness, predicting treatment response, and guiding personalized therapy. In a recently published systematic review [22], the HI showed variable levels of correlation with pathological characteristics and the prediction of tumor staging across a wide range of cancers (breast, head and neck, esophagus, etc.) Although promising results were obtained for predicting treatment failure and tumor relapse, there is no consensus on how HI should be calculated, and its role in oncology remains unclear. In our cohort of patients, no significant heterogeneity was observed in the radiopharmaceutical distribution within the lesions, suggesting that BCC might have less biological variability compared to other tumor types, potentially leading to a more uniform treatment response. Nonetheless, further research is needed to determine whether HI has a significant prognostic role in BCC.

Regarding the use of ^18^F-FDG PET/CT in evaluating treatment response, the only similar published study, by Thacker et al., assessed response to vismodegib using PET/CT [10]. They found that a 33% reduction in total SUVmax during treatment correlated with longer progression-free survival (PFS) and overall survival (OS) compared to those who did not achieve this reduction. Notably, they applied both PERCIST and EORTC (European Organization for Research and Treatment of Cancer) criteria for response assessment.

Both the PERCIST and EORTC criteria aim to standardize tumor response assessment using PET/CT, but they differ in approach [23,24,25,26]. The EORTC criteria, being older, use the mean SUV of the tumor for evaluation and classify responses into categories such as complete metabolic response, partial metabolic response, stable metabolic disease, and progressive metabolic disease. However, EORTC criteria are less stringent about scanning conditions, which can introduce variability. PERCIST, a newer set of guidelines, focuses on SUVpeak within a small tumor volume and emphasizes consistent scanning conditions, such as fasting blood glucose levels and post-injection timing, to ensure reliable measurements. This specificity helps reduce variability and enhances the precision of metabolic response assessment. In our cohort, we applied PERCIST due to its more detailed and standardized approach compared to the broader EORTC criteria [27]. Our findings demonstrated that the PERCIST aligned with clinical evaluations. Although the follow-up period was limited, no relapse or progression was observed in the 6 months following the second PET/CT scan, suggesting that PET/CT-detected metabolic changes may be indicative of a durable clinical response.

Additionally, considering the timing of PET/CT evaluations after initiating sonidegib therapy is important. Given that the action of HPIs is generally slow, as observed by Thacker et al., who reported a median response time of 183 days, we conducted evaluations with an 8-month follow-up [28].

The main limitations of this study are represented by the small sample size, which constrains the generalizability of the results, the single-center characteristic, and the relatively short study duration. In addition, in our cohort of patients, following a multidisciplinary consensus meeting, ¹⁸F-FDG PET/CT was identified as the preferred imaging approach for evaluating patients with advanced BCC at baseline and after HPI. Consequently, as contrast-enhanced CT was not routinely performed, response evaluation according to the Response Evaluation Criteria in Solid Tumors (RECIST) was not available and should be the subject of further investigation.

Advanced BCC is rare, and clinical experiences with sonidegib are often based on small cohorts. In a recent single-center study of 19 patients, 15 (78.9%) experienced a benefit, although no correlation was found between clinicopathological and treatment characteristics [29]. Another issue is determining when to discontinue HPI treatment. A recent multicenter study of 68 BCC patients treated with vismodegib suggested that discontinuing HPI therapy after achieving a complete clinical response may improve disease-free survival [30]. However, this has not yet been thoroughly investigated in patients receiving sonidegib. Further research is needed to explore the role of PET/CT in decisions about discontinuing HPI therapy.

It should be underlined that the use of PET/CT in BCC cannot be considered routine, also taking into account the cost/benefit balance. These tumors are primarily located in the head/neck region and rarely tend to metastasize. In our center, we have reserved the use of PET/CT for patients with advanced BCC with the aim of acquiring prognostic information and monitoring the response to a recently implemented therapy, allowing for a whole-body assessment to identify potential sites of distant progression as well as to detect the metabolic correlations of any adverse reaction. ^18^F-FDG PET/CT shows promise in managing advanced BCC by providing accurate assessments of the disease’s metabolic burden and treatment response, which can help optimize therapeutic strategies. The potential applications of PET/CT in oncology, especially in dermato-oncology, remain underexplored. Advances in radiopharmaceuticals, such as Fibroblast Activation Protein ligands, and the development of ‘long axial field-of-view’ PET/CT scanners with extraordinary sensitivity could revolutionize its use in both oncological and non-oncological fields [31,32]. Additionally, radiomics, which involves extracting reproducible quantitative data from morphological and functional images, is yielding promising results for developing predictive models of oncological outcomes [33].

## 5. Conclusions

Our findings indicate that ^18^F-FDG PET/CT might be utilized to assess treatment response in patients with locally advanced BCC undergoing sonidegib therapy. A baseline evaluation of the disease’s metabolic characteristics may help identify patients who are more likely to benefit from targeted therapy with HPI. Further research is required to better define the role of metabolic imaging in this clinical setting, considering recent advancements in radiopharmacy and PET technology.

## Figures and Tables

**Table 1:** The clinical and demographic characteristics of the selected patients.

Pt/Sex	Age	Comorbidities	Previous Therapies	Histotype	Dermatoscopy	Location/Number of Lesions
1, F	74	Depression, diabetes	None	Nodular	Small ulcerations, telangiectasias, arborizing vessels	Ear, bilateral face n = 3
2, M	85	Hypertension, BHP	None	Nodular, invasive	Telangiectasias, arborizing vessels, blue-gray ovoid nests	Face, n = 3
3, M	77	Hypertension, BHP, UP	PDT	Nodular, ulcerative	Telangiectasias, arborizing vessels, ulceration	Ear, bilateral n = 2
4, M	89	Hypertension	ECT	Nodular, invasive	Telangiectasias, arborizing vessels, blue-gray ovoid nests	Ear, bilateral n = 2

Pt: patient; F: female; M: male; BHP: benign prostatic hyperplasia; ECT: electrochemotherapy; UP: urothelial papilloma; PDT: photodynamic therapy.

**Table 2:** Metabolic parameters and response to HPI in the selected patients.

Pt	PET Response	SUVmax	SUVmean	HI	MTV (cc)	TLG (g/mL)
1	CMR	6.5	2.9	2.2	1.3	3.7
2	CMR	5.1	3.2	1.6	0.5	1.6
3	SMD	9.1	4	2.2	4.4	17.9
4	SMD	16.9	10.8	1.5	1	11.8
		9.4 ± 4.5, 7.8 *	5.2 ± 3.2, 3.6 *	1.8 ± 0.3, 1.9 *	1.8 ± 1.7, 1.1 *	8.7 ± 6.5, 7.7 *

Pt: patient; SUV: standardized uptake value; HI: heterogeneity index; MTV: metabolic tumor; TLG: total lesion glycolysis; * mean ± standard deviation, median.

## Data Availability

Original data are available from the corresponding authors for reasonable motivations.

## Supplementary Materials

The following supporting information can be downloaded at: https://www.mdpi.com/article/10.3390/jcm13175087/s1, Supplemental Data S1.
