# Supplementary material for: Metabolic Imaging of Advanced Basal Cell Carcinoma Treated with Sonidegib: A Retrospective Case Series Study
Source: J Clin Med. 2024 Aug 27;13(17):5087. doi: 10.3390/jcm13175087 (PMC11396429; doi:10.3390/jcm13175087)
Supplement: Supplementary file 1 [file jcm-13-05087-s001.zip › jcm-3131142-supplementary.pdf]

Supplemental data 1

PET/CT scan acquisition protocol and image reconstruction

A CT scan covering the area from the upper thighs to the skull base was performed with a 1.0 mm slice thickness, a pitch factor of 1, and reconstruction kernels for both bone and soft tissue. The scan was conducted with a maximum of 120 keV and 90 mAs, employing CARE kV and CARE Dose technologies. After the CT scan, a whole-body PET (from the upper thighs to the skull vertex) was captured 3-5 minutes post tracer administration in 3D (matrix: 440x440) with a zoom factor of 1.0. The digital PET scan was acquired using a Siemens Biograph Vision 450 with a 197 mm axial FOV, utilizing continuous bed motion (FlowMotion®) at a speed of 0.9 mm/s, which is roughly equivalent to 2 minutes per bed position. Reconstruction was performed using the TrueX + TOF algorithm and was Gauss-filtered to achieve a transaxial resolution of 2 mm at FWHM (full width at half maximum). Attenuation correction was based on the low-dose, non-enhanced CT data.
